# Perioperative Variables Contributing to the Rupture of Intracranial Aneurysm: An Update

**DOI:** 10.1155/2013/396404

**Published:** 2013-11-12

**Authors:** Tumul Chowdhury, Ronald B. Cappellani, Nora Sandu, Bernhard Schaller, Jayesh Daya

**Affiliations:** ^1^Department of Anesthesiology, Health Sciences Center, University of Manitoba, 2nd Floor Harry Medovy House, 671 William Avenue, Winnipeg, MB, Canada R3E 0Z2; ^2^Research, University of Southampton, University Road, Southampton S017 1 BJ, UK

## Abstract

*Background*. Perioperative aneurysm rupture (PAR) is one of the most dreaded complications of intracranial aneurysms, and approximately 80% of nontraumatic SAHs are related to such PAR aneurysms. The literature is currently scant and even controversial regarding the issues of various contributory factors on different phases of perioperative period. Thus this paper highlights the current understanding of various risk factors, variables, and outcomes in relation to PAR and try to summarize the current knowledge. *Method*. We have performed a PubMed search (1 January 1991–31 December 2012) using search terms including “cerebral aneurysm,” “intracranial aneurysm,” and “intraoperative/perioperative rupture.” * Results*. Various risk factors are summarized in relation to different phases of perioperative period and their relationship with outcome is also highlighted. There exist many well-known preoperative variables which are responsible for the highest percentage of PAR. The role of other variables in the intraoperative/postoperative period is not well known; however, these factors may have important contributory roles in aneurysm rupture. Preoperative variables mainly include natural course (age, gender, and familial history) as well as the pathophysiological factors (size, type, location, comorbidities, and procedure). Previously ruptured aneurysm is associated with rupture in all the phases of perioperative period. On the other hand intraoperative/postoperative variables usually depend upon anesthesia and surgery related factors. Intraoperative rupture during predissection phase is associated with poor outcome while intraoperative rupture at any step during embolization procedure imposes poor outcome. *Conclusion*. We have tried to create such an initial categorization but know that we cannot scale according to its clinical importance. Thorough understanding of various risk factors and other variables associated with PAR will assist in better clinical management as well as patient care in this group and will give insight into the development and prevention of such a catastrophic complication in these patients.

## 1. Introduction

Subarachnoid hemorrhage (SAH) is one of the most devastating neurological diseases. This condition not only produces the significant impact on mortality and morbidity but also imparts dire social consequences [1–3]. Perioperative aneurysm rupture (PAR) is one of the most dreaded complications of intracranial aneurysms and approximately 80% of nontraumatic SAHs are related to such ruptured intracranial aneurysms. PAR will depend upon the natural course of disease, contributory risk factors, and time of intervention [4]. PAR will be influenced by different variables presented during the preoperative, intraoperative, and postoperative period. However, the literature is currently scant and even controversial regarding the issues of the various contributory factors during the different phases of the perioperative period. Thus this paper highlights the current understanding of various risk factors, variables, and outcomes in relation to the perioperative rupture of intracranial aneurysm and try to summarize the current knowledge.

## 2. Method

We have performed a PubMed search (1 January 1991–31 December 2012) using search terms including “cerebral aneurysm,” “intracranial aneurysm,” and “intraoperative/perioperative rupture.” Only papers in the English language that specifically discussed the relevant complication and various risk factors were included. The articles related to complications and management of complex giant aneurysms and pediatric aneurysm were not included in this review.

## 3. Risk Factors for Aneurysm Rupture and Outcome

The mechanism of cerebral aneurysmal rupture remains unknown at present. It is, however, known that a chronic inflammatory reaction is occurring within the aneurysmal wall, being associated with the degeneration of the aneurysmal wall and susceptibility of the aneurysm to bleeding [5]. However, screening or identification of bleeding-prone cerebral aneurysms cannot yet be performed, so that the knowledge of the various risk factors and variables is important and can be mainly divided into the three phases of the perioperative period. There exist many well-known preoperative variables which are responsible for the highest percentage of aneurysm rupture. The role of other variables in the intraoperative as well as postoperative period is not well known; however, these factors may have important contributory roles in aneurysm rupture.

### 3.1. Preoperative Variables

Though the overall risk of rupture in unruptured cerebral aneurysm is low (<0.5% per year) except for giant aneurysms, the morbidity and mortality associated with rupture can be high [6]. These unruptured cerebral aneurysms often remain asymptomatic, or sometimes, the smaller aneurysms can produce also compressive symptoms (involvement of cranial nerves mainly third and forth) or ischemic symptoms due to thromboembolic episodes [7, 8]. Thus, it is imperative to know about the natural course (age, gender, and familial history) as well as the pathophysiological factors (size, type, location, comorbidities, procedure) which would govern cerebral aneurysm rupture perioperatively [9]. In addition, postrupture of cerebral aneurysm certain preventable medical conditions further adds to overall morbidity and mortality. In addition, one study has highlighted that comorbidities (such as arterial hypertension, congestive heart failure, and electrolyte disturbances) were associated with increase in the likelihood of longer median length of stay in all patients with cerebral aneurysms [10, 11].

Prevalence of unruptured cerebral aneurysms is increasing worldwide and is estimated to be approximately 0.65%–3.2%. The two most common risk factors associated with SAH are patients with adult polycystic kidney (ADPKD) and a positive family history of intracranial aneurysm of SAH [12]. Interestingly, in some countries like Japan and Finland, higher incidence of SAH is not found to be correlated with higher prevalence of unruptured aneurysms (UIA) and thus suggests the existence of other cofactors responsible for causing SAH in this population [13].

Female gender represents also a significant risk factor. However, the influence of female hormones on the pathogenesis of aneurysm formation and rupture is inconclusive. In the affected families, the most frequent relationship exists between the siblings. The genetic predisposition is a significant contributory factor for multiplicity as well as location of aneurysm. In addition, familial SAH was found to be associated with larger aneurysm at the time of rupture [13, 14].

A Japanese study group revealed that more than 90% of cerebral aneurysm cases were incidentally detected and warrants the need for preventive as well as screening methods to detect unruptured aneurysm. Most common aneurysms detected in this study were in the middle cerebral arteries (MCA) and the internal carotid arteries (ICA); however, aneurysms located in anterior (ACoA) and posterior communicating artery (PCoA) were more likely to be ruptured in comparison to MCA aneurysms [15]. Familial intracranial aneurysm (FIA) study also showed the preponderance of MCA location as opposed to International Study of Unruptured Intracranial Aneurysms (ISUIA) which had more patients with PCoA aneurysms (patients who had a family history of intracranial aneurysm or subarachnoid hemorrhage were excluded) [14]. The effect of geographical area on incidence and type of aneurysm rupture is inconclusive at present; however, Japanese study showed that the large, posterior-circulation, and symptomatic aneurysms were associated with significantly higher rates of rupture. The selection bias could not be eliminated from this study and could possibly account for the higher incidence of rupture. In contrast, US based study found that more than 90% of the unruptured aneurysms were located in the anterior circulation and a location was also similar for patients with ruptured aneurysms. However, the lack of correlation of size and age at rupture (exposure to risk factors) suggests that the size at rupture is more dependent on hemodynamic stress [16, 17].

According to one of the largest studies, age is the most significant risk factor associated with surgical outcome; however, the size and location of an aneurysm influence both surgical as well as endovascular outcomes [18]. Similarly, the International Study of Unruptured Intracranial Aneurysms (ISUIA) found that a higher rupture rate was reported to be associated with larger aneurysms, located in the posterior circulation, and previous history of SAH.

Biochemical factors linked together with ICA rupture were also investigated but not well understood. Complement dysregulation is the major mechanism which in turn increases the susceptibility to complement activation, inflammation, and tissue damage within the intra-arterial wall. The role of gene variations of endothelial nitric oxide synthase (eNOS), a vasomodulatory protein to assist in identifing patients with a high likelihood of rupture, has also been highlighted [19, 20]. Apoptosis is the described major mechanism responsible for the process of aneurysm rupture.

The geometrical as well as biophysical characteristics were also studied for the future prediction of cerebral aneurysm rupture and include but are not limited to three-dimensional diameter (D-max), aspect ratio, flow angle, parent-daughter angle, wall shear stress, and oscillatory shear index (OSI). However, none of these factors have been found to be superior to one another [21–23]. The predictors of aneurysm growth were also studied and included as a diameter of at least 10 mm and a location at the basilar artery (BA) bifurcation or the ICA [24].

Some of the rare types of cerebral aneurysms also have an effect on the periprocedural rupture rate and thus increase the overall morbidity and mortality. In this regard, intrasellar aneurysms can impose grave complications. Most of them are associated with either compressive symptoms or endocrinopathy and often mimic as pituitary tumors [25, 26]. The other rarer type of aneurysm often described as blood-blister like aneurysm was also very notorious to be ruptured acutely and often requires internal carotid artery trapping combined with high-flow extracranial-intracranial (trapping/EC-IC) bypass during the acute period following SAH [27–30]. The management of this type of aneurysm is often challenging for both the surgeon and interventional neuroradiologist. Pericallosal aneurysms, which comprise another rarer group, often have an increased chances of intraoperative rupture [26].

The role of diurnal variation on cerebral aneurysm rupture has not been well studied; however, nocturnal rupture associated SAH has been found to be an independent risk factor for developing cerebral ischemia and poor outcome [31]. The relation between sexual intercourse and chances of aneurysm rupture was also highlighted in a review and could be one of the possibilities of higher incidence of aneurysm rupture among young individuals [32]. 

Cerebral aneurysms which are present at bifurcations tend to be more at risk for rupture due to shear wall stress; however, in the study of 77 PCoA aneurysms, true PCoA aneurysms might be more prone for rupture than junctional aneurysms of similar size [33]. Similarly lower risks were observed for the rupture of carotid bifurcation aneurysms and further support the hypothesis that the anatomical geometry of the bifurcations and concomitant hemodynamic stress are not the sole factors which govern the overall risk of rupture [34].

### 3.2. Intraoperative Variables

#### 3.2.1. Preprocedural Factors

Though rare, the preprocedural rupture of cerebral aneurysms can produce catastrophic complications with high mortality. Anesthesia related factors contribute to this phase of intraoperative rupture. There exists a fine balance between mean arterial pressure, ICP and transmural pressure (TMP). The acute changes in hemodynamics during the periods of induction, surgical incision, and skull pin fixation may increase the transmural pressure and may be responsible for intraoperative aneurysm rupture [35, 36]. Sudden coughing or gagging during any point of time can evoke acute rise in blood pressure and intracranial pressure that, hence, leads to rupture.

Raised intracranial pressure (ICP) is usually associated with poor grade SAH but may present in good grade too. Raised ICP can again affect the TMP and can precipitate intraoperative aneurysm rupture (IOAR). However, data do not support significant link between raised ICP and intraoperative rupture of aneurysm [37]. The other variables like rapid boluses of mannitol and excessive hyperventilation before the dural opening may produce IOAR [35].

#### 3.2.2. Intraprocedural Factors

The intraprocedural rupture (IPAR) mainly depends upon vessel wall fragility which in turn may be modified by the several comorbidities including coronary artery disease, hyperlipidemia, race, COPD, and lower Hunt and Hess grade (Table 1) [38].

In study of 1010 patients with cerebral aneurysm surgery (299 coiled, 711 clipped), 14.6% developed intraprocedural aneurysm rupture (IPAR) (19% with clipping and 5% with coiling). There was significant higher mortality in coiling related IPAR [38]. Predissection phase variables which are responsible for IOAR usually include dural opening and arachnoid opening, hematoma removal, and brain retraction. In study of 398 patients with ruptured aneurysm, 6% showed premature (before securing the parent vessel) rupture of aneurysm and significantly contributed to brain swelling [39]. The partial resection of brain could give the space for temporarily clipping and could be found to be related to poor neurological outcome. In another study in 170 patients with ACA and MCA rupture aneurysm, there was 9% incidence of intraoperative rupture due to the procedure itself. Intraoperative aneurysmal rupture usually occurred during dissection of the aneurysm, dissection of the artery adhering to the aneurysm, or during clip application [40].

In a retrospective study of 1269 surgically treated patients, 113 IOAR events occurred. The posteroinferior cerebellar artery and ACoA and PCoA aneurysms were more liable to rupture intraoperatively. The IOR rate was greater in ruptured than unruptured aneurysms [41].

Experience of the surgeon certainly plays a crucial role during the intraprocedural period. Data suggests that increase in surgical experience reduces the number of intraprocedural ruptures, duration of temporary clipping, and the surgical mortality rate and hence favors better outcome [42]. This conclusion also exists for the neurointerventional surgeon/radiologist. The association between the types of surgical approaches and intraoperative rupture requires further attention and is described in more detail elsewhere.

Neurovascular management of very small aneurysms often imposes challenges for the both the surgical as well as interventional procedures [43]. Interestingly in one study small cerebral aneurysms were associated with larger volume of SAH; however the other factors including patient sex and age, intraparenchymal or intraventricular hemorrhage, multiple aneurysms, history of hypertension, and aneurysm location were not associated with a larger volume of SAH [44, 45]. The chances of intraprocedural aneurysm rupture were found to be relatively higher during embolization especially in very small aneurysms. The main causes were coil protrusion and microcatheter perforation. Angiographic demonstration of deterioration in cerebral hemodynamics was found to be an independent detriment of poor outcome. The study on endovascular management (CLARITY trial) revealed that a higher proportion of patients with intraoperative rupture were found to be younger (age less than 65 year) as well as hypertensive and more commonly associated with MCA aneurysm [46]. Furthermore, authors concluded that it is the location of aneurysm not the size which predicts the chances of intraoperative aneurysm rupture. On the other hand, the same study highlighted the significance of both location and size of aneurysm neck in causing thrombo-embolic complications. Another study revealed that there were 5 times greater chances of intraprocedural (endovascular) rupture in previously ruptured very small aneurysm in comparison with large ruptured aneurysm (Table 2). In this regard, balloon assisted hemostasis was associated with a better outcome. Furthermore, when coiling alone was compared with remodeling methods, the intraprocedural ruptures were found to be similar with comparable overall mortality and morbidity [47, 48].

On the other hand, management of very large or even giant cerebral aneurysms is also very complex and associated with significant risk [29]. Surgical indications are usually reserved for younger patients; however, depending upon risk and benefit ratio, elderly age group patients warrant considerations for endovascular management. Minimization of temporary occlusion and the use of intraoperative angiography have been shown to favor the surgical outcome in these cases [49].

The present literature is also variable regarding the intraoperative rupture and long-term neurological outcome. Some studies have concluded that intraoperative rupture adversely affects the outcome; however, these data should be interpreted in relation to various factors including size, location, procedure (clipping versus coiling), age, management strategies, and experience of surgeon [50]. The outcome is usually worse in cases of IOR associated with coiling. Even in the hybrid suite, the intraoperative rupture during coiling was associated with 40% mortality and 20% long-term morbidity. On the other hand, outcome was found to be better after IOR of small aneurysm during coiling in comparison with a large one [51].

Temporarily arterial occlusion at the time of the clipping is now well-established modality, and still may impose many complications. In a retrospective study of 112 patients, 17% of the patients were found to have symptomatic stroke and 26% had radiological evidence of stroke attributable to temporary arterial occlusion [52]. Advancing age and poor clinical grade were the risk factors significantly associated with symptomatic stroke. Intraoperative cerebral aneurysm rupture and duration of temporary clip application that lasted more than 20 minutes were found to be independent predictors of perioperative stroke outcome. In this regard, duration of single clipping seems to be more important risk factors rather than the placement of multiple clips for the short period of time [53].

### 3.3. Postoperative Variables

There are only few reports available regarding the postoperative cerebral aneurysm rupture in which residual aneurysm or arterial dissection was highlighted, and the factors contributed were intracranial hypotension due to excessive CSF drainage, hemodynamic stress caused by hypertension, and trauma due to surgical manipulation [54, 55].

There have been reports of aneurysm recurrence and subsequent SAH from small residual necks (1-2mm) after clipping [56]. On the other hand, in cases of incomplete occlusion after coiling, this may impose more chances of rerupture risks; however, the data is scant at present so the contributory factors are yet to be studied.

## 4. Future Directions

To really better understand the risk factors that influence cerebral aneurismal rupture, we need first to better understand the mechanism of rupture. Imaging methods may assist us to gain better insights into these underlying mechanisms that are clinically important. It is surprisingly that until now very few relevant studies have been performed in this context. Such created knowledge would assist to better categorize the numerous risk factors that are currently known and discussed within this paper. We have tried to create such an initial categorization (Table 3) but know that we cannot scale according to its clinical importance. However, deeper analysis shows that these situations are anything but simple and are often additionally influenced by dogmas in treatment that makes it not easier to compare the treatment between different institutions. But to go ahead a further step includes openly describing also errors and adverse effects that have been seen during the treatment of cerebral aneurysm, a fact that lacks generally in medicine.

In conclusion, thorough understanding of various risk factors and other variables associated with perioperative aneurysm rupture will assist in better clinical management as well as patient care in this group and will give insight into the development and prevention of such a catastrophic complication in these patients.

## Figures and Tables

**Table 1:** Intraoperative variables contributing intracranial aneurysm rupture.

	Outcome
*(1) Preprocedural factors (same for coiling procedures) *	
Anesthesia related	Poor
Pain	
Light plane of anesthesia	
Intubation response	
Acute fluctuations in TMP	
High blood pressure (induction, skull pin fixation, and incision)	
Coughing/gagging	
Raised ICP	
Large and rapid bolus mannitol	
Acute hyperventilation	
*(2) Intraprocedural factors *	Variable
Increased vessel wall fragility (same for coiling procedures)	Poor
Smoking/COPD	
Hyperlipidemia/CAD	
Lower Hunt and Hess grade	
Predissection phase	Poor
(Dural opening and arachnoid opening, hematoma removal, and brain retraction)	
Dissection phase	Variable
Type of artery (PICA, ACA, and PCA)	
Previously ruptured aneurysm	
Surgeon's experience	

**Table 2:** Intraoperative variables contributing intracranial aneurysm rupture (coiling only).

	Outcome
Preprocedural	
Hypertensive patients	Poor
Angiography in acute phase of SAH	
Contrast injection induced hypertension	
Intraprocedural factors	Poor
Age younger than 65 years	
Very small aneurysms	
Wide neck > 4 mm	
Type of artery (MCA and posterior circulation)	
Previously ruptured aneurysm	
Experience of neuroradiologist	

**Table 3:** Perioperative variables contributing intracranial aneurysm rupture.

Perioperative variable	Outcome
*(1) Preoperative factors *	Variable
Age	
Female gender	
Size (large aneurysm) and location (posterior circulation aneurysm)	
Genetic factors/familial factors	
Hypertension	
Smoking	
Ruptured aneurysm	Poor
*(2) Intraoperative factors *	Poor
Preprocedural factors	
Anesthesia related	
Factors affecting TMP	
Predissection variables	
Intraprocedural variables	
Predissection variables	Poor
Dissection variables	
Coiling versus surgery	Poor/variable
Size and location	
Comorbidities	
*(3) Postoperative factors *	Poor
Emergence/extubation related	
Surgical dissection/trauma	
Incomplete obliteration by coiling or clipping	
